# Identifying driver pathways based on a parameter-free model and a partheno-genetic algorithm

**DOI:** 10.1186/s12859-023-05319-8

**Published:** 2023-05-23

**Authors:** Jingli Wu, Qinghua Nie, Gaoshi Li, Kai Zhu

**Affiliations:** 1 Key Lab of Education Blockchain and Intelligent Technology, Ministry of Education, Guangxi Normal University, Guilin, China; 2 Guangxi Key Lab of Multi-source Information Mining and Security, Guangxi Normal University, Guilin, China; 3 College of Computer Science and Engineering, Guangxi Normal University, Guilin, China

**Keywords:** Cancer, Driver pathway, Protein–Protein interaction, Partheno-genetic algorithm

## Abstract

**Background:**

Tremendous amounts of omics data accumulated have made it possible to identify cancer driver pathways through computational methods, which is believed to be able to offer critical information in such downstream research as ascertaining cancer pathogenesis, developing anti-cancer drugs, and so on. It is a challenging problem to identify cancer driver pathways by integrating multiple omics data.

**Results:**

In this study, a parameter-free identification model SMCMN, incorporating both pathway features and gene associations in Protein–Protein Interaction (PPI) network, is proposed. A novel measurement of mutual exclusivity is devised to exclude some gene sets with “inclusion” relationship. By introducing gene clustering based operators, a partheno-genetic algorithm CPGA is put forward for solving the SMCMN model. Experiments were implemented on three real cancer datasets to compare the identification performance of models and methods. The comparisons of models demonstrate that the SMCMN model does eliminate the “inclusion” relationship, and produces gene sets with better enrichment performance compared with the classical model MWSM in most cases.

**Conclusions:**

The gene sets recognized by the proposed CPGA-SMCMN method possess more genes engaging in known cancer related pathways, as well as stronger connectivity in PPI network. All of which have been demonstrated through extensive contrast experiments among the CPGA-SMCMN method and six state-of-the-art ones.

## Introduction

Cancer, a disease with high mortality, is generally caused by the mutation of driver genes [1–4]. Different from passenger ones, whose mutations are irrelevant to cancers, the mutations of driver genes promote the infinite proliferation and spread of cancer cells [5]. Previous studies have demonstrated that the difficulty of diagnosing and treating cancers is attributed to enormous mutational heterogeneity inherent in cancer genomes. That is to say, there are many significant cellular signaling transduction pathways or regulatory ones responsible for cell proliferation, metabolism and apoptosis [6, 7]. Each of them possesses a group of driver genes. The mutation on any one of these driver genes is generally sufficient to disturb the regulatory function of a pathway and result in cancers. Therefore, the identification of a group of driver genes enriched in a pathway, i.e., driver pathway, is essential for studying the pathogenic mechanism of cancers. Since it is time-consuming as well as expensive to identify through biological experiments in the lab, it is a very economic way to detect driver pathways (driver gene sets) by applying computational approaches on the abundant accumulated multi-omics data. This has received widely concern in bioinformatics [8–10].

There are generally two kinds of methods to identify cancer driver pathways: de novo methods and prior knowledge-based ones. The de novo methods attempt to discover a set of genes, having two fundamental features of driver pathways such as high coverage and high mutual exclusivity, by using just genetic data. High coverage means that the gene mutations in one driver pathway cover abundant cancer samples, while high mutual exclusivity indicates that any two genes in one pathway seldom mutate in the same cancer sample. Based on such two features, Vandin et al. [9] firstly proposed the maximum weight submatrix model trying to minimize both coverage and mutual exclusivity in 2012, and solved it with a markov chain monte carlo based method Dendrix (De novo Driver mutual exclusivity). Later, Zhao et al. [11] put forward the binary linear programming method and the GA (Genetic Algorithm) one to solve the model. Both of which exhibit better performance than the Dendrix method, and the GA method is particularly convenient to solve the integrative model incorporating the gene expression profiles. In 2013, Zhang et al. [12] integrated two weighted networks constructed from mutation matrix and expression one, and proposed a network-based approach iMCMC (identify Mutated Core Modules in Cancer) to extract core modules from the integrated network. A module with specified size can not be produced by this method. In 2016, based on the GA method, method MOGA was devised to balance the trade-off between coverage and mutual exclusivity [13]. In 2017, Yahya et al. [14] put forward the QuaDMutEx method, which identifies gene sets through adopting monte carlo optimization and binary quadratic programming. In 2019, Wu et al. [15] improved the maximum weight submatrix model and proposed method PGA-MWS for solving this problem. In 2021, Wu et al. [10] introduced a weighted non-binary mutation matrix. They formulated a new maximum weight submatrix model by redefining coverage and mutual exclusivity, and devised a cooperative co-evolution algorithm CGA-MWS for solving this model. In most cases, algorithm CGA-MWS can identify a gene set possessing more genes involving in one known signaling pathway compared with previous methods.

In the above de novo methods, mutation frequency based pre-filtering is usually conducted to decrease the number of combinations of genes. Hence, some pathways containing rare mutations may be ignored [16]. Prior knowledge-based methods regard genes with high mutation rates and their less-frequently mutated neighbors as drivers, and attempt to detect them from known gene-level or protein-level pathways or networks [17], such as MEXCOwalk [16], HotNet [18], IDM-SPS [19] and HotNet2 [20]. However, biological networks are still associated with noise and incomplete. The intuition of combining these two kinds of methods, i.e., taking advantage of fundamental features of a driver pathway and gene relationships in biological networks, has germinated. In 2020, Yahya et al. [17] presented method QuaDMutNetEx, which is extended from their QuaDMutEx method by incorporating the connectivity of genes in the identification model. Experimental results indicate that method QuaDMutNetEx can identify some driver genes with low mutation rate compared with method QuaDMutEx. The integration of driver pathway features and prior knowledge does work.

**Fig. 1:**
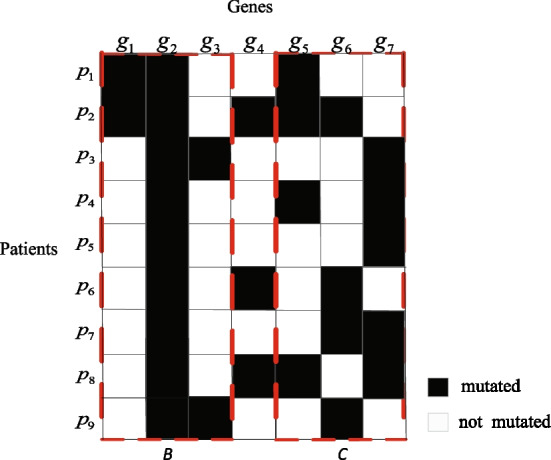
An example of mutation matrix

Among the above mentioned identification methods, some parameters need to be preset to adjust the weight of different omics data, such as methods iMCMC [12], MOGA [13], QuaDMutEX [14], PGA-MWS [15], and QuaDMutNetEx [17]. This may limit their usability and scalability, for an large number of experiments are usually required to ascertain these parameters before applying them. Moreover, the identification model, adopted in such methods as Dendrix [9], GA [11], MOGA [13], may not distinguish two gene sets with exact different coverage or mutual exclusivity in some cases. As shown in Fig. 1, there is a mutation matrix with rows representing a set of cancer samples, and columns representing a set of genes. The black entries indicate genes mutate in the corresponding samples, while white ones otherwise. Between gene sets *B* and *C*, although gene sets *C* is expected to be selected for its genes having more uniform distribution in coverage than *B*, they are not able to be differentiated in terms of the maximum weight submatrix model (the weight function values of *B* and *C* are equal to 5) used in methods Dendrix, GA and MOGA.

Therefore, a measurement of mutual exclusivity, excluding some gene sets with “inclusion” relationship (e.g. gene set B in Fig. 1), is studied. An identification model without preset parameters is studied from the perspective of combining driver pathway features with prior knowledge in biological networks. The main contributions of the article include:

1. A novel relative hamming distance RHD is devised for calculating the distance between a gene and a gene set. Hence, given a gene set, the average RHD value between each gene and the rest genes measures the mutual exclusivity of the set.
2. An identification model SMCMN, which is parameter free, is formulated by exploring a Submatrix with Maximum Coverage, mutual exclusivity and Network connectivity.
3. The CPGA algorithm, based on gene clustering and partheno-genetic algorithm, is proposed. Novel operators are devised to initialize and mutate individuals in terms of gene clustering.
4. Real cancer datasets were applied to test the performance of the presented CPGA-SMCMN method, and compare it with six state-of-the-art ones.

## Methods

### Definitions and notations

Suppose there are a somatic mutation matrix $S_{\vert P\vert \times \vert G \vert }$, and a copy number variation matrix $C_{\vert P \vert \times \vert G \vert }$. The rows and columns of them denote the same cancer sample set *P* and gene set *G*, respectively. Each entry $s_{ij}$ $\in${0,1} (*i* = 1,2,...,$\vert P \vert$, *j* = 1,2,...,$\vert G \vert$) of matrix *S* indicates whether the *j*th gene mutates in the *i*th sample or not. In matrix *C*, $c_{ij}$ = ± 1 (*i* = 1,2,...,$\vert P \vert$, *j* = 1,2,...,$\vert G \vert$) means the *j*th gene is in a statistically significant variation region of the *i*th sample, and $c_{ij}$ = 0 otherwise. In addition, two matrices $F_{\vert G \vert \times \vert G \vert }$ and $E_{\vert G \vert \times \vert G \vert }$ record the correlation between genes, where $f_{ij}$ of matrix *F* denotes the relationship extracted from the literature, and $e_{ij}$ of matrix *E* denotes the one obtained from experiments (*i*, *j* = 1,2,...,$\vert G \vert$). Each entry of them ranges from 0 to 999, and are normalized into the range between 0 to 1.

Construct matrices *S* and *C* into a binary mutation matrix $A_{\vert P \vert \times \vert G\vert }$. Entry $a_{ij}$ (*i* = 1,2,...,$\vert P \vert$, *j* = 1,2,...,$\vert G \vert$) equals to 1 if and only if both $s_{ij}$ and $c_{ij}$ are not equal to 0 simultaneously, and 0 otherwise. A new correlation matrix $W_{\vert G \vert \times \vert G \vert }$ is also generated by combining matrices *F* and *E*, where $w_{ij}$(*i*, *j* = 1,2,...,$\vert G\vert$) is ascertained as Equation (1):

1 $$\begin{aligned} w_{ij} = {\left\{ \begin{array}{ll} \max \{f_{ij},e_{ij}\}, &{} \text{ if } \,e_{ij}\ne 0,\\ 0, &{} \text{ otherwise }. \end{array}\right. } \end{aligned}$$

**Fig. 2:**
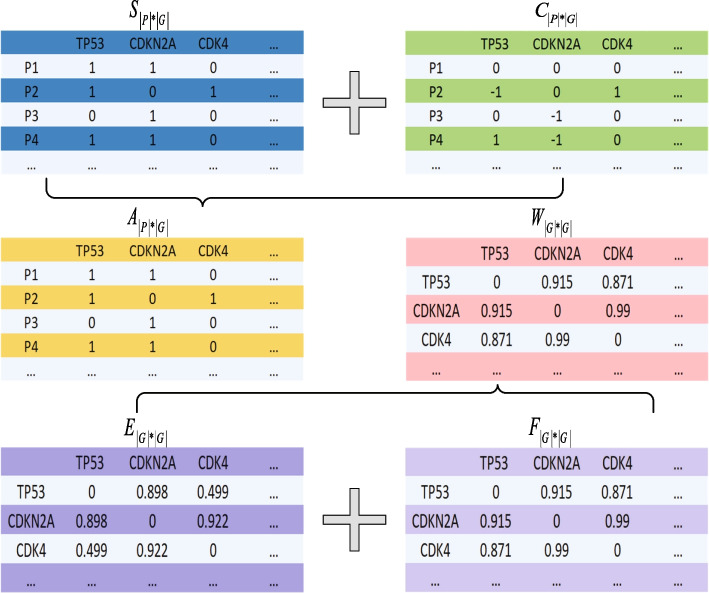
Schematic diagram of constructing binary mutation matrix *A* and correlation matrix *W*

Fig. 2 shows the schematic diagram for constructing matrices *A* and *W*.

Let $\Gamma (g_j)$ = {$a_{i-}$ $\vert$ $a_{ij}$ $=$1, $g_j$ $\in$ $G$} (*i* = 1, 2, ..., $\vert P\vert$) record the set of samples in which gene $g_j$ mutates. Given any $\vert P\vert$ $\times$ $K$ submatrix of *A*, denoted by *M*, let $\Gamma (M)$ = $\bigcup _{a_{-j}\in M}\Gamma (g_j)$ represent the set of samples in which the genes of matrix *M* mutate. As shown in Fig. 1, *B* and *C* are a pair of submatrices with *K* = 3. They have the same weight in terms of formula $2\vert \Gamma (M)\vert$ $-$ $\sum _{a_{-j}\in M}{\vert \Gamma (g_j)\vert }$ (*M* denotes a mutation submatrix), which is adopted by methods Dendrix [9], GA [11] and MOGA [13]. Nevertheless, it is apparently that in submatrix *B*, all of the patients mutating on genes $g_1$ and $g_3$ are covered by those mutating on gene $g_2$, we call gene $g_2$ “includes” genes $g_1$ and $g_3$, i.e., there are two “inclusion” relationships in gene set *B*. Hence submatrix *C* is expected to be selected for its genes having more uniform distribution in coverage than those of submatrix *B*. Since submatrices *B* and *C* can not be distinguished exactly well in terms of the above weight function, a new measurement is devised in this study.

Let *CO*(*M*) measure the “coverage” of matrix *M*, i.e., the ratio of samples covered by matrix *M* to the total mutation ones:

2 $$\begin{aligned} CO(M)=\frac{\vert \Gamma (M)\vert }{\max \{\vert \Gamma (g_j)\vert \vert g_j\in G\}}, \end{aligned}$$

where *G* records the set of genes in matrix *A*. Given a pair of genes $g_j$ and $g_k$ of mutation matrix *A* ($g_j$, $g_k$ $\in$ $G$), let *RHD*($g_j$,$g_k$) represent the Relative Hamming Distance between $g_j$ and $g_k$, i.e., the hamming distance of gene $g_j$ relative to gene $g_k$, as Formula (3):

3 $$\begin{aligned} RHD(g_j,g_k)=\frac{\sum _{i=0}^{\vert P \vert }{d(a_{ij},a_{ik})}}{\vert \Gamma (g_j)\vert }, \end{aligned}$$

where *d*($a_{ij}$,$a_{ik}$) is defined as Formula (4):

4 $$\begin{aligned} d(a_{ij},a_{ik})={\left\{ \begin{array}{ll} 1, &{}\text{ if } \,a_{ij}=1 \;and \;a_{ik}=0,\\ 0, &{}\text{ otherwise }. \end{array}\right. } \end{aligned}$$

For submatrix $M_{\vert P\vert \times K}$ in matrix *A*, let *RHD*($g_j$,*M*) denote the Relative Hamming Distance between gene $g_j$ and gene set $G_M$ $\setminus${$g_j$} ($G_M$ denotes the set of genes in *M*, $g_j$ $\in$ $G_M$):

5 $$\begin{aligned} RHD(g_j,M)=\frac{\sum _{g_k\in G_M\setminus {g_j}}RHD(g_j,g_k)}{K-1}. \end{aligned}$$

Take matrix *B* in Fig. 1 for an example. *RHD*($g_1$, $g_2$) = 0, *RHD*($g_2$, $g_1$) = $\frac{7}{9}$, *RHD*($g_1$, $g_3$) = 1, *RHD*($g_3$, $g_1$) = 1, *RHD*($g_2$, $g_3$) = $\frac{7}{9}$, *RHD*($g_3$, $g_2$) = 0, *RHD*($g_1$, *M*) = $\frac{1}{2}$, *RHD*($g_2$, *M*) = $\frac{7}{9}$, *RHD*($g_3$, *M*) = $\frac{1}{2}$. Then the “mutual exclusivity” *ME*(*M*) can be measured as the average *RHD* between each gene of *M* and the rest genes of it. Greater *ME*(*M*) denotes higher mutual exclusivity of matrix *M*. In Fig. 1, the same result 5 will be obtained when calculating matrices *B* and *C* using the formula $2\vert \Gamma (M)\vert$ $-$ $\sum _{a_{-j}\in M}{\vert \Gamma (g_j)\vert }$, it is difficult to distinguish gene set *B* from *C*. However, they are easy to be distinguished with Formula (6), for *ME*(*B*) = $\frac{16}{27}$, and *ME*(*C*) = $\frac{83}{120}$. The obvious choice is matrix *C* for its larger *ME*(*M*).

6 $$\begin{aligned} ME(M) = \frac{\sum _{g_j\in G_M}RHD(g_j,M)}{K}. \end{aligned}$$

In addition, let *N*(*M*) indicate the correlation among the genes in matrix *M*, as shown in Formula (7):

7 $$\begin{aligned} N(M) = \frac{\sum _{i=1}^{\vert G_{M}\vert }\sum _{j=1}^{\vert G_{M}\vert }\widetilde{w}_{ij}}{\vert G_{M}\vert \times (\vert G_{M}\vert -1)}, \end{aligned}$$

where $\widetilde{w}_{ij}$ denotes the entry of matrix $\widetilde{W}_{\vert G_M\vert \times \vert G_M\vert }$, which is a submatrix extracted from the correlation matrix *W*.

Based on the above definition, a combinatorial model SMCMN, ascertaining a submatrix with Maximum Coverage, mutual exclusivity, and Network connectivity, is established: given a mutation matrix $A_{\vert P\vert \times \vert G\vert }$, a correlation matrix $W_{\vert G\vert \times \vert G\vert }$, and a parameter *K* (0 $K$ $\vert G\vert$), identify a submatrix $M_{\vert P\vert \times K}$ to maximize the weight function *W*(*M*):

8 $$\begin{aligned} \begin{aligned} W(M)&=CO(M)+ME(M)+N(M)\\&=\frac{\vert \Gamma (M)\vert }{\max \{\vert \Gamma (g_j)\vert \vert g_j\in G\}}+\frac{\sum \nolimits _{g_j\in G_M}RHD(g_j,M)}{K}+\frac{\sum \nolimits _{i=1}^{\vert G_{M}\vert }\sum \nolimits _{j=1}^{\vert G_{M}\vert }\widetilde{w}_{ij}}{\vert G_{M}\vert \times (\vert G_{M}\vert -1)}. \end{aligned} \end{aligned}$$

### CPGA-SMCMN algorithm

In this part, an algorithm based on gene clustering and Partheno-Genetic Algorithm (we name it as CPGA) is put forward for solving the SMCMN model. The input is a binary mutation matrix *A*, a correlation matrix *W*, and a parameter *K* (0 $K$ $\vert G\vert$). The output is a submatrix *M*. The key steps of the CPGA-SMCMN method are described at first, and then the pseudo code of it is illustrated.

#### Clustering preprocessing

As indicated in the previous section, the intrinsic computational complexity of this problem owes to a large number of combinations of mutated genes. Therefore, in the preprocessing stage, two gene clusters are built for each gene, so as to drop some combinations of genes with weak correlations in advance. Given gene $g_j\in G$, let $c_1(g_j)$ record the set of genes that have greater relative hamming distance with gene $g_j$, i.e., $c_1(g_j)$ = {$g_k$ $\vert$ $RHD$($g_j$,$g_k$)$\ge$ $\mu$, $g_k$ $\in$ $G$ $-${$g_j$}}. Similarly, $c_2(g_j)$ is constructed to record the set of genes that have greater correlation with gene $g_j$, i.e., $c_2(g_j)$ = {$g_k$ $\vert$ $w$(*j*,*k*)$\ge$ $\nu$, $g_k$ $\in$ $G$ $-${$g_j$}}. Here $\mu$ and $\nu$ are two preset parameters.

#### Individual representation and population

The representation of a solution is generally used to encode an individual, i.e., a chromosome. In the CPGA-SMCMN method, a chromosome is encoded by a set of *K* genes, i.e., $X$ $=${$x_1$,$x_2$,...,$x_K$} ($x_j$ $\in${1, 2, ..., $\vert G\vert$}, $j$ $=$1, 2, ..., *K*). *N* chromosomes construct a population. The initialization of a chromosome is depicted as the following two steps:

1. Select a gene $g_j$ ($g_j$ $\in$ $G$) with roulette strategy, i.e., greater $\vert \Gamma (g_{j})\vert$ contributes to higher probability of choosing gene $g_{j}$. Let $X$ $=${*j*}.
2. Iteratively select the rest $K$ $-$1 genes with roulette strategy. Assume that $X$ $=${$x_{1}$,$x_{2}$,...,$x_{k}$} (1$\le$ $k$ $K$). Let $\widetilde{C}$ $=$ $\bigcap _{j = 1}^k$ $c_1(g_{x_j})$ $\cap$ $\bigcup _{j = 1}^k$ $c_2(g_{x_j})$. The next gene $g_r$ ($g_{r}$ $\in$ $\widetilde{C}$) is chosen in terms of $\frac{\vert \Gamma (g_r)\vert }{\sum _{t = 1}^{\vert \widetilde{C}\vert }\vert \Gamma (g_{y_t})\vert }$ ($y_t$ $\in${1,2,...,|*G*|}), and should meet the constraint of $\frac{\sum _{t = 1}^{k} w(g_r, g_{x_t})}{k}$ $\ge$ $\nu$ ($\nu$ is a preset parameter). A chromosome can not be created successfully if $\widetilde{C}$ $=$ $\emptyset$ at any one iteration.

#### Fitness function

Fitness measures the viability of individuals in a population, and pilots the direction of evolution. Given chromosome *X*, let $M_X$ represent a $\vert P\vert$ $\times$ $K$ submatrix of *A*, the columns of $M_X$ come from the genes in *X*. Then weight function $W(M_X)$ is adopted to evaluate the fitness of chromosome *X*, as defined in Equation (9). The greater *Fitness*(*X*) is, the more viable the solution *X* is.

9 $$\begin{aligned} Fitness(X) = W(M_X) \end{aligned}$$

#### Genetic operators

In a partheno-genetic algorithm, selection operator and recombination one are required to generate offspring. In the CPGA algorithm, both roulette wheel selection and elitist strategy are adopted, i.e., an individual with higher fitness has a higher probability of being selected, and the individual with the highest fitness will be remained during the process of evolution. Furthermore, a greedy-based recombination operator is devised so as to enhance the population diversity, and escape from premature convergence as well as the local optimum, as follows:

1. Given chromosome *X* = $\{x_1,x_2,\dots ,x_K\}$ ($x_j$ $\in${1,2,...,$\vert G\vert$}, *j* = 1,2,...,*K*), one of the following two methods is executed randomly to drop a gene from *X*. 1) Drop the gene from *X* that mutates in the fewest patients, i.e., $x_j$ = $\mathop {\arg \min }\limits _{x_j\in X}\vert \Gamma (g_{x_j})\vert$, 2) Drop a gene from *X* randomly. The new chromosome is denoted by $\widehat{X}$.
2. Let $\widetilde{C}$ $=$ $\bigcap _{x_j\in \widehat{X}}$ $c_1(g_{x_j})$, select the gene $g_r$ ($g_{r}$ $\in$ $\widetilde{C}$) having the largest $\vert \Gamma (g_r)\vert$ on the premise of meeting $\frac{\sum _{t = 1}^{k} w(g_r, g_t)}{k}$ $\ge$ $\nu$ ($\nu$ is a preset parameter). If there is no eligible gene found from $\widetilde{C}$, chromosome *X* remains unchanged.

#### CPGA-SMCMN

The CPGA-SMCMN method is described in Algorithm 1. In Step 1, some parameters used in this method are set. Step 2–4 implement preprocessing, taking time $O({|G|}^{2}|P|)$. In Step 5, the generation of an initial population of size *N* takes time *O*(*NK*|*P*||*G*|). Step 6 initializes the best individual, and the calculation of fitness takes time $O(N|P|(K^2+|G|))$. The entire evolution, controlled by *maxg* and *maxt*, is performed from Step 7 to Step 20, where roulette wheel selection and recombination operators are executed iteratively from Step 9 to Step 14, taking time *O*(*maxgNK*|*G*||*P*|). Finally the best individual is translated and output in Step 21 and 22. Therefore, the total maximum time complexity of algorithm CPGA-SMCMN is $O(|G||P|(|G|+maxgNK))$.
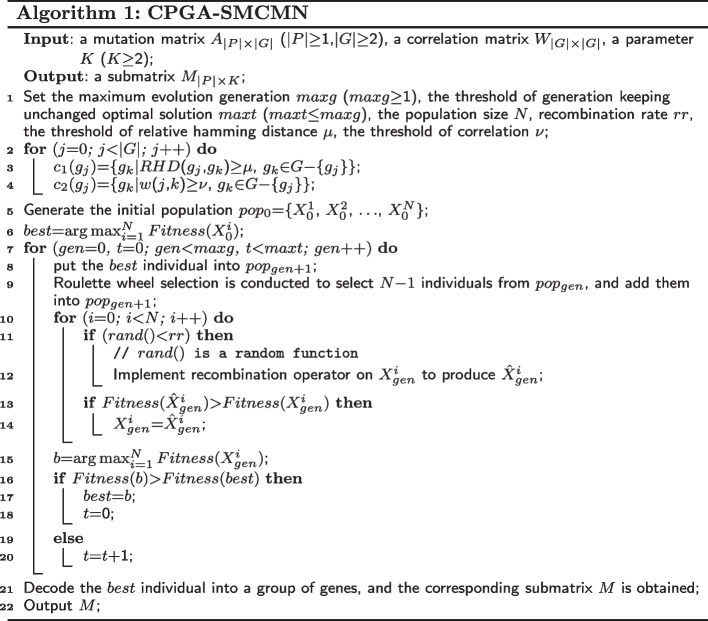


## Results

In this section, experimental comparisons are conducted based on real biological data. We begin by testing the models which are based on the proposed coverage and mutual exclusivity, and comparing them with the famous one proposed by Vandin et al. [9], which has also been used in such methods as Dendrix [9], GA [11], and MOGA [13]. Then the identification performance of method CPGA-SMCMN was compared with six state-of-the-art methods, i.e., Dendrix [9], CGA-MWS [10], GA [11], iMCMC [12], MOGA [13] and PGA-MWS [15]. The experimental comparisons were implemented on a Lenovo PC with Intel(R) Core(TM) i7-7700 3.60GHz CPU and 24GB RAM. The operating system was Windows 11, the compiler used by the Dendrix, the CPGA and the CPGA-SMCMN methods is Python 3.0 in PyCharm 2018.1.4, the compiler used by the GA and the CGA-MWS methods is MyEclipse 2016 CI, the compiler used by the PGA-MWS method is R x64 4.1.0.

### Experimental dataset

Three sorts of cancer datasets were adopted in the experiments: glioblastoma (GBM), ovarian cancer (OVCA), and thyroid cancer (THCA). The mutation data of glioblastoma and ovarian cancer were get from Zhao et al. [11]. The mutation data of thyroid cancer and the copy number variation data of the three cancers were obtained from TCGA (http://tcga-data.nci.nih.gov/tcga/). GISTIC [21] was applied to transfer the value of the copy number variation data from its original one to $-1$, 0, or 1. The association confidence values among genes, which respectively comes from the literature and experiments, were acquired from the STRING network (https://cn.string-db.org/).

In the three datasets, the genes which mutate in less than 0.5$\%$ samples were dropped. In addition, since Gene *TP53* are very prevalent (mutating in more than 80% of samples, much higher than other genes mutating in less than 25% of samples) and *TTN* may be artifacts in the OVCA dataset, they are deleted from the dataset [11]. Table 1 shows the processed data, where column “Edges” indicates the number of edges among the corresponding genes in the STRING network.

**Table 1:** The experimental data of three sorts of cancers

Cancers	Patients	Genes	Edges
GBM	90	920	55686
OVCA	313	2547	414682
THCA	487	1613	151714

### Parameter setting and evaluation index

The parameters of the CPGA-SMCMN method were set as follows: *N* = $\frac{\vert G\vert }{4}$, *maxg* = 1000, *maxt* = 100, *rr* = 0.3, $\mu$ = 0.7, $\nu$ = 0.5, which were ascertained through a large number of experimental tests, as shown in Appendix. The Dendrix method was executed for $10^6$ iterations and sampled a set every $10^3$ iterations. The parameters of method GA were set as: *maxg* = 1000, *maxt* = 10, *N* = $\vert G\vert$, and $P_m$ = 0.1. The ones of method PGA-MWS were set as: *maxg* = 500, *maxt* = 10, *N* = $log_{2}(\prod \nolimits _{i = 0}^{K-1}\vert G \vert -i$), $\alpha$ = 0.7, $\beta$ = 10, and $\tau$ = $\frac{\vert G\vert }{5}$. The ones of method CGA-MWS were set as: *maxg* = 1000, *maxt* = 10, *N* = $\frac{\vert G\vert }{4}$, $P_m$ = 0.3, $\lambda _1$ = 3, and $\lambda _2$ = 7. The gene sets detected by methods iMCMC and MOGA were directly referred to literature [12, 13], for their source codes were not acquired.

In the experiments, pathway enrichment as well as network connectivity are adopted to evaluate the identified gene sets. That is to say, given a detected gene set, the more genes enriched in a cancer-related biological pathway, the better the gene set is. Similarly, it is also anticipated that more genes of the set connect in the PPI network. The cancer related pathways used in the analysis and discussion of experimental results are referred to the KEGG database (http://www.genome.jp/kegg/).

A random test [12] was employed to calculate the significance of the identified gene sets. Given a submatrix *M* with *K* detected genes, its significance is calculated as Formula (10):

10 $$\begin{aligned} \textit{p}-value = \frac{\sum \nolimits _{i = 1}^{1000}W(M_i)> W(M)}{1000}, \end{aligned}$$

where $M_i$ denotes a submatrix with *K* randomly selected genes.

### Comparison of models

In this section, experiments were performed to evaluate the pathway enrichment of the gene sets acquired under different identification models, i.e., the proposed models and the one proposed by Vandin et al. [9]. The models were solved with the same parthenogenetic algorithm of method PGA-MWS [15]. Tables 2, 3 and 4 show the comparison results on such three cancer datasets as GBM, OVCA and THCA. MWSM denotes the Maximum Weight Sub-Matrix model proposed by Vandin et al. [9], SMCM and SMCMN denote two models based on the proposed coverage and mutual exclusivity, while model SMCM indicates the one that does not consider the network connectivity. The genes displayed in bold means that they are engaging in the same cancer-related pathway. Moreover, let $r_{pe}$ indicate the percentage of genes enriched in the same signaling pathway among the identified genes. It has the same meaning in the subsequent tables.

**Table 2:** Pathway enrichment of gene sets under different models (GBM dataset)

*K*	MWSM	$r_{pe}(\%)$
2	**CDKN2B CDK4**	100.0
3	**CDKN2B CDK4 RB1**	100.0
4	**CDKN2B RB1** *TSPAN31 ERBB2*	50.0
5	**CDKN2B RB1** *ERBB2 TSPAN31 PPP2R1A*	40.0
6	**CDKN2B RB1 CDK4** * ERBB2 MSH2 NKG7*	50.0
7	**CDKN2B RB1 CDK4** *DBC1 BCAS1 CD33 ERBB2*	42.9
8	**ERBB2 CDK4 RELN FGF21** * RB1 PRF1 NTRK3 CDKN2B*	50.0

*K*	SMCM	$r_{pe}(\%)$
2	**CDKN2B CDK4**	100.0
3	**CDKN2B CDK4 TP53**	100.0
4	**CDKN2B CDK4 RB1 TP53**	100.0
5	**FGFR3 NF1 TP53** *CDKN2B TSPAN31*	60.0
6	**NF1 TP53** * CDKN2B CYP27B1 DBC1 SYNE1*	33.3
7	**EGFR TP53 CDK4 RELN TEK** *CDKN2B NF1*	57.1
8	**EGFR TP53 FGFR3 RELN** *NF1 CDKN2B SYNE1 CYP27B1*	50.0

*K*	SMCMN	$r_{pe}(\%)$
2	**CDKN2B CDK4**	100.0
3	**CDKN2A CDK4 TP53**	100.0
4	**CDKN2A CDK4 TP53 RB1**	100.0
5	**CDKN2A CDK4 TP53 CCNE1 RB1**	100.0
6	**PIK3CA TP53 PTEN ERBB2 EGFR** *CDKN2A*	83.3
7	**PIK3CA TP53 PTEN ERBB2 EGFR PIK3R1** *CDKN2A*	85.7
8	**CDKN2A CDK4 TP53 CASP3 CCNE1** *RB1 PIK3CA FOXO1*	62.5

Bold indicate that the genes are enriched in the same biological signaling pathway

**Table 3:** Pathway enrichment of gene sets under different models (OVCA dataset)

*K*	MWSM	$r_{pe}(\%)$
2	**MYC CCNE1**	100.0
3	**MYC CCNE1** *NINJ2*	66.7
4	**MYC CCNE1** *NINJ2 ABCC10*	50.0
5	**MYC CCNE1** * COL5A3 NINJ2 ABCC10*	40.0
6	**MYC CCNE1** *COL5A3 NINJ2 MYH4 ABCC10*	33.3
7	**MYC CCNE1** *COL5A3 NINJ2 MYH4 ABCC10 TRAPPC8*	28.6
8	**MYC CCNE1** *COL5A3 NINJ2 MYH4 ABCC10 TRAPPC8 PRPC7*	25.0

*K*	SMCM	$r_{pe}(\%)$
2	**MYC CCNE1**	100.0
3	**MYC CCNE1 KRAS**	100.0
4	**MYC CCNE1** *NINJ2 MACF1*	50.0
5	**MYC CCNE1** *NINJ2 MACF1 NF1*	40.0
6	**MYC CCNE1** *NINJ2 MACF1 NF1 ARFRP1*	33.3
7	**MYC CCNE1** * NINJ2 MACF1 NF1 MBD3 ZNF512B*	28.6
8	**MYC CCNE1 PPP2R2A** *NINJ2 MACF1 NOTCH3 TPD52L2 RYR2*	37.5

*K*	SMCMN	$r_{pe}(\%)$
2	**MYC CCNE1**	100.0
3	**MYC KRAS CCNE1**	100.0
4	**MYC KRAS CCNE1** *FBXW7*	75.0
5	**MYC KRAS CDH1 CTNNB1** *CCNE1*	80.0
6	**MYC KRAS CDH1 CTNNB1** *CCNE1 NOTCH3*	66.7
7	**MYC KRAS CDH1 CTNNB1** *CCNE1 NOTCH3 FZD2*	57.1
8	**MYC KRAS CCNE1 PTEN NRAS** *NF1 BRAF NOTCH3*	62.5

Bold indicate that the genes are enriched in the same biological signaling pathway

**Table 4:** Pathway enrichment of gene sets under different models(THCA dataset)

*K*	MWSM	$r_{pe}(\%)$
2	**BRAF NRAS**	100.0
3	**BRAF NRAS HRAS**	100.0
4	**BRAF NRAS HRAS PTEN**	100.0
5	**BRAF NRAS HRAS** *GLUD1 CNTLN*	60.0
6	**BRAF NRAS HRAS** *LIPJ CNTLN ZCCHC2*	50.0
7	**BRAF NRAS HRAS** *SLC25A45 CNTLN DOCK6 PRKG1*	42.9
8	**BRAF NRAS HRAS** *GLUD1 CNTLN DOCK6 SLC1A6 SLC25A45*	37.5

*K*	SMCM	$r_{pe}(\%)$
2	**BRAF NRAS**	100.0
3	**BRAF NRAS HRAS**	100.0
4	**BRAF NRAS HRAS** *MRPS16*	75.0
5	**BRAF NRAS HRAS PTEN** *CNTLN*	80.0
6	**BRAF NRAS HRAS** *TDRD7 DOK5 IFIT3*	50.0
7	**BRAF NRAS HRAS** *EIF3L SLC25A45 RUFY2 ABHD16A*	42.9
8	**BRAF NRAS HRAS** *TG ZCCHC2 ZNF385D PRKG1 SEC14L2*	37.5

*K*	SMCMN	$r_{pe}(\%)$
2	**BRAF NRAS**	100.0
3	**BRAF NRAS HRAS**	100.0
4	**BRAF NRAS HRAS RAF1**	100.0
5	**BRAF NRAS HRAS PTEN** *PIK3CG*	80.0
6	**BRAF NRAS HRAS PIK3CA KRAS RAF1**	100.0
7	**BRAF NRAS HRAS PIK3CA PTEN** *PIK3R5 MUC16*	71.4
8	**BRAF NRAS HRAS PIK3CA PTEN KRAS** *TP53 DIS3*	75.0

Bold indicate that the genes are enriched in the same biological signaling pathway

From Tables 2, 3 and 4, we can notice that in most cases, based on models SMCM and SMCMN, the identification algorithm is able to acquire gene sets which have more genes involving in one known cancer related pathway. As shown in Table 2, except for *K* = 6, the number of enriched genes based on model SMCM is greater than or equal to that based on model MWSM. The gene sets detected based on model SMCMN possess more genes engaging in one known cancer related pathway than those identified based on model MWSM under each *K* setting. In addition, it is noticed that when *K* = 7, there exactly exists an “inclusion” relationship in the gene set acquired by model MWSM, i.e., the samples mutating on gene *CD33* are covered utterly by those mutating on gene *CDK4*. The genes obtained by models SMCM and SMCMN do exempt from the relationship. In Table 3, although there is no apparent difference in the number of enriched genes detected based on models MWSM and SMCM, the number of which identified based on model SMCMN is apparent greater than that based on model MWSM. In Table 4, except for *K* = 4 and 5, the gene sets recognized based on models MWSM and SMCM have the same number of genes enriched in one cancer related pathway. Model SMCMN still performs the best among the three models in terms of the number of enriched genes under each *K* setting. Therefore, the proposed coverage and mutual exclusivity play a positive effect on optimizing identification, and the introduction of network connectivity further improves the ability of optimization.

### Comparison of methods

In this section, experiments were conducted to compare the identification performance of methods Dendrix [9], GA [11], iMCMC [12], MOGA [13], PGA-MWS [15], CGA-MWS [10] and CPGA-SMCMN. In addition, the performance of algorithm CPGA for solving the classical MWSM model was also tested and presented.

#### Glioblastoma

**Fig. 3:**
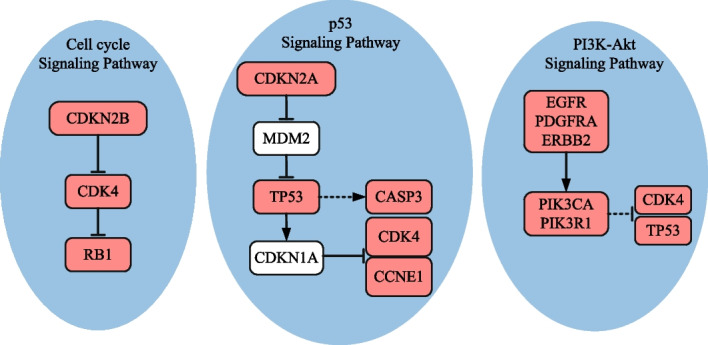
Biological pathways enriched with the genes detected by method CPGA-SMCMN (GBM dataset). The solid line represents a direct interaction between two genes, and the dotted one indicates an indirect one. The pink nodes denote the genes detected by method CPGA-SMCMN. The same notations are used in the subsequent figures

Table 5 compares the identification results under different *K* settings. When *K* = 2, each detected gene set, except for (*CDKN2A, CYP27B1*) identified by method iMCMC, is enriched in one cancer-related biological pathway. Specifically, gene set (*CDKN2B, CDK4*), detected by methods GA, PGA-MWS, CGA-MWS, CPGA, and CPGA-SMCMN, enriches in the *cell cycle* signaling pathway (Fig. 3). It was declared that the *cell cycle* and the *MAPK* signaling pathways may be disturbed simultaneously and cooperatively involved in the initiation and progression of GBM [22]. Gene set (*CDKN2A, TP53*), detected by methods Dendrix and MOGA, enriches in the *p53* signaling pathway. When *K* = 3, except for methods iMCMC and MOGA, the other six methods can produce a gene set engaged in one cancer-related pathway. In terms of the KEGG database, gene set (*RB1, CDKN2B, CDK4*) detected by methods Dendrix, GA, and CPGA is part of the *cell cycle* signaling pathway, and gene set (*CDKN2A, TP53, CDK4*) detected by methods PGA-MWS, CGA-MWS, and CPGA-SMCMN is part of the *p53* signaling pathway (Fig. 3). The deregulated *p53* signaling pathway is generally discovered in GBM, and its components are related to GBM cell invasion, migration, proliferation, escape from apoptosis and cancer cell stem cells [23].

**Table 5:** Comparisons of experimental results on the glioblastoma dataset

*K*	Dendrix	*Time*(*s*)	$r_{pe}(\%)$
2	**CDKN2A TP53**	94.2	100.0
3	**RB1 CDKN2B CDK4**	101.7	100.0
4	**RB1 CDKN2B CDK4** *FUT2*	104.5	75.0
5	**RB1 CDKN2B CDK4** *FGFR1 CARD8*	128.1	60.0
6	**RB1 CDKN2B** *COL4A1 GML PIH1D1 TSPAN31*	125.5	33.3
7	**RB1 CDKN2B** *CYP27B1 GPR19 PPP1R115A PAN1 TAS2R9*	125.5	28.6
8	**RB1 CDKN2B** *CCNE1 CHEK1 CSNK2A2 NOVA2 PDE6H TSPAN31*	128.0	25.0

*K*	GA	*Time*(*s*)	$r_{pe}(\%)$
2	**CDKN2B CDK4**	5.5	100.0
3	**CDKN2B CDK4 RB1**	6.0	100.0
4	**CDKN2B CDK4 RB1** *ERBB2*	6.2	75.0
5	**CDKN2B CDK4 RB1** *ERBB2 EMP3*	6.2	60.0
6	**CDKN2B CDK4 RB1** *ERBB2 CSF1R FCGRT*	6.1	50.0
7	**CDKN2B CDK4 RB1** *ERBB2 FGFR3 NTRK3 ROR2*	6.3	42.9
8	**CDKN2B CDK4 RB1** *ERBB2 FGFR3 NTRK3 ROR2 SPHK2*	6.3	37.5

*K*	iMCMC	*Time*(*s*)	$r_{pe}(\%)$
2	*CDKN2A CYP27B1*	–	–
3	**TP53 PTEN** *MTAP*	–	–
4	**EGFR MDM2** *NF1 CHAT*	–	–

*K*	MOGA		
2	**CDKN2A TP53**	–	–
3	**CDKN2B CDK4** *TP53*	–	–

*K*	PGA-MWS	*Time*(*s*)	$r_{pe}(\%)$
2	**CDKN2B CDK4**	6.0	100.0
3	**CDKN2A CDK4 TP53**	11.0	100.0
4	**CDKN2A CDK4 TP53** * NF1*	24.0	75.0
5	**CDKN2A CDK4 TP53** *COL6A3 NF1*	39.0	60.0
6	**CDKN2A CDK4 TP53** *COL6A3 NF1 SHH*	42.0	50.0
7	**CDKN2A CDK4 TP53** *COL6A3 NF1 SHH TSPAN31*	101.0	42.9
8	**TP53 COL6A3 COL6A2** *CDKN2B NF1 RCBTB2 TSPAN31 SHH*	110.0	37.5

*K*	CGA-MWS	*Time*(*s*)	$r_{pe}(\%)$
2	**CDKN2B CDK4**	0.5	100.0
3	**CDKN2A CDK4 TP53**	0.5	100.0
4	**CDKN2B CDK4 RB1** *TP53*	0.7	75.0
5	**CDKN2B CDK4 RB1** *TP53 EGFR*	0.8	60.0
6	**CDKN2B CDK4 RB1** *TP53 EGFR DBC1*	0.8	50.0
7	**CDKN2B CDK4 RB1** *TP53 EGFR DBC1 NTRK3*	1.0	42.9
8	**TP53 CDK4 EGFR FGFR3** *CDKN2B RB1 NTRK3 DBC1*	0.8	50.0

*K*	CPGA	*Time*(*s*)	$r_{pe}(\%)$
2	**CDKN2B CDK4**	14.9	100.0
3	**CDKN2B CDK4 RB1**	14.0	100.0
4	**CDKN2A CDK4** *RB1 ERBB2*	14.7	50.0
5	**CCNE1 CDK4 ERBB2** *CDKN2B RB1*	22.7	60.0
6	**CCNE1 CDK4 ERBB2 TP53** *CDKN2B RB1*	25.5	66.7
7	**CCNE1 CDK4 ERBB2 TP53 FGFR3** *CDKN2A RB1*	26.7	71.4
8	**TP53 MDM2 EGFR** *CASP3 PRKDC CDH1 CTNNB1 NUMB*	30.2	37.5

*K*	CPGA-SMCMN	*Time*(*s*)	$r_{pe}(\%)$
2	**CDKN2B CDK4**	30.5	100.0
3	**CDKN2A TP53 CDK4**	14.1	100.0
4	**CDKN2A TP53 CDK4** *RB1*	8.1	75.0
5	**CDKN2A TP53 CDK4 CCNE1** *RB1*	8.1	80.0
6	**CDKN2A TP53 CDK4 CCNE1** *ERBB2 RB1*	7.9	66.7
7	**CDKN2A TP53 CDK4 CCNE1 CASP3** *ERBB2 RB1*	22.2	71.4
8	**TP53 CDK4 EGFR ERBB2 PDGFRA PIK3R1 PIK3CA** *CTNNB1*	15.0	87.5

Bold indicate that the genes are enriched in the same biological signaling pathway

When *K* = 4–8, the number of enriched genes found by method CPGA-SMCMN is equal to or greater than those identified by the other methods. With the increase of *K*, the advantage becomes more and more obvious. When *K* = 4–7, the identified gene sets (*CDKN2A, TP53, CDK4*) and (*CDKN2A, TP53, CDK4, CCNE1, CASP3*) are involved in the *p*53 signaling pathway. When *K* = 8, there are seven genes (*TP53, CDK4, EGFR, ERBB2, PDGFRA, PIK3R1, PIK3CA*) involving in the *PI3K-Akt* signaling pathway. It was regarded that the *PI3K/Akt/mTOR* pathway is implicated to growth, survival, metabolism, autophagy, angiogenesis, and chemotherapy resistance of GBM [24]. Among genes (*RB1*, *ERBB2*, *CTNNB1*) identified by method CPGA-SMCMN, although they are not enriched in a biological pathway with any other identified genes, they have been reported to be important cancer related genes. For example, the retinoblastoma *RB1* gene is a tumor suppressor one, whose status is identified as a determinant of glioblastoma therapeutic efficacy [25]. *ERBB2* has been implied as an appropriate target for *CAR T* cells in glioblastoma, its expression is often associated with high-grade gliomas [26]. It has been discovered that the expression of *CTNNB1* was substantially higher in $IDH1^{WT}$ gliomas than in $IDH1^{MUT}$ one, indicating that it is probable for gene *CTNNB1* to have a correlation with immunosuppressive microenvironment [27]. In addition, compare the results of methods CPGA and Dendrix, which apply different identification algorithms on the maximum weight submatrix model. The results indicate that the proposed partheno-genetic algorithm exhibits stronger optimization ability than the markov chain monte carlo algorithm used in method Dendrix.

**Fig. 4:**
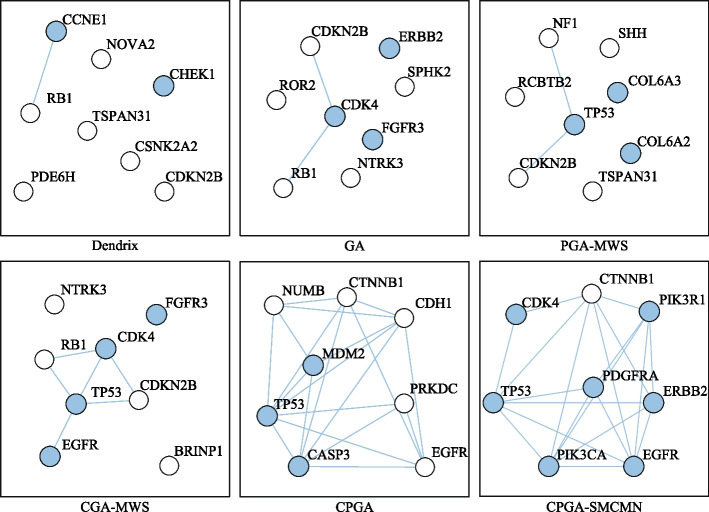
Connectivity of genes in the PPI network (GBM dataset, *K* = 8)

**Fig. 5:**
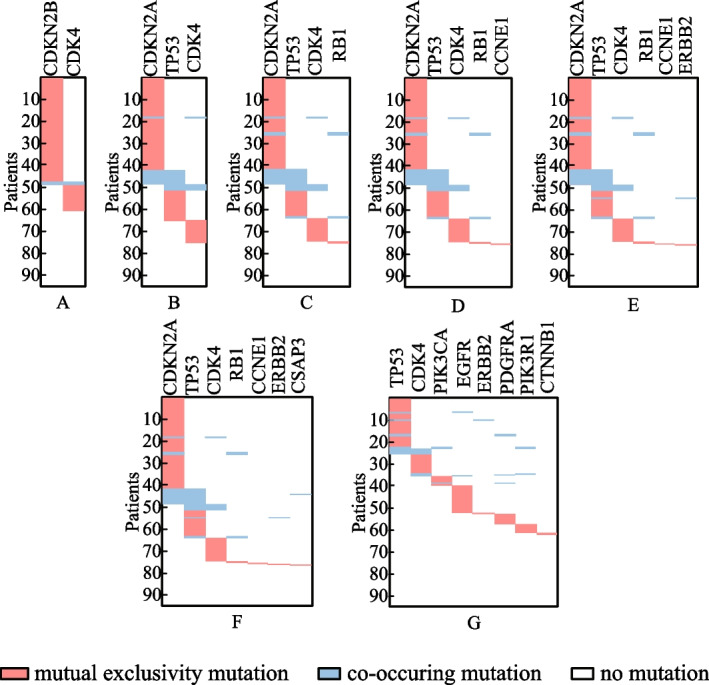
The coverage and mutual exclusivity of the gene sets detected by the CPGA-SMCMN method (GBM dataset)

Besides the identified gene sets, the execution efficiency is also compared among these identification methods. The running time of methods iMCMC and MOGA was not presented (denoted by −), for their source codes were not acquired. As shown in Table 5, all of the methods can execute with relatively high efficiency. Figure 4 exhibits the connectivity of genes identified by different methods in the PPI network for *K* = 8. The genes engaging in one cancer related pathway are labeled in blue. It can be noticed that the genes obtained by methods CPGA-SMCMN and CPGA manifest better connectivity than those identified by other methods. The seven gene sets acquired by the CPGA-SMCMN method were subjected to significance tests, and each of them has a *p-value* of less than 0.005. Furthermore, their coverage and mutual exclusivity are illustrated in Fig. 5, where mutual exclusivity mutations are denoted by red bars, co-occurring mutations are denoted by blue bars, and no mutations are denoted by white bars. It is apparent that all of the seven gene sets show preferable coverage and mutual exclusivity. More than two-thirds of patients are covered by each gene set. Genes *CDKN2B* and *CDKN2A* mutate in more than half of patients, respectively. It has been validated that *CDKN2A/B* deletion is a prognostic biomarker for IDH-wildtype GBM [28]. In addition, several low-frequency mutation genes were detected by method CPGA-SMCMN and were involved in the same pathway with other detected genes. For example, gene *PIK3CA* mutates in 5 samples, and gene *PIK3R1* mutates in 6 samples. They were all missed by other contrast methods.

#### Ovarian carcinoma

**Fig. 6:**
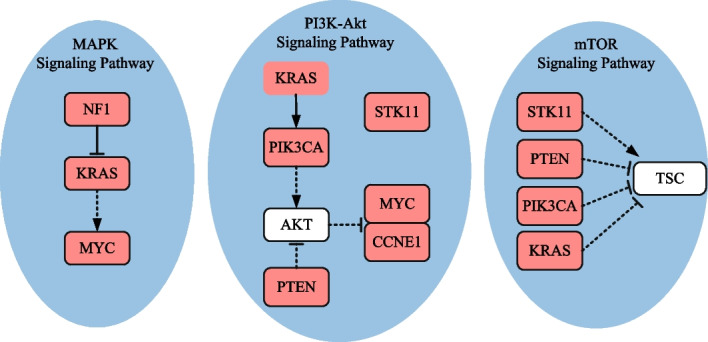
Biological pathways enriched with the genes detected by method CPGA-SMCMN (OVCA dataset)

Table 6 compares the identified gene sets as well as execution efficiency based on the ovarian carcinoma dataset, where *K* = 2–8. Since Zheng et al. [13] had not provided the identified gene sets on ovarian cancer dataset, the MOGA method is not compared in Table 6. When *K* = 2, except for method iMCMC, each of the methods produces a gene set engaging in the *PI3K-Akt* or the *MAPK* signaling pathways (Fig. 6). It has been reported that the *PI3K-Akt* signaling pathway is a critical one for therapeutic intervention in ovarian cancer [29]. The *MAPK* signaling pathway is a critical regulator of ovarian cancer cell proliferation [30].

**Table 6:** Comparisons of experimental results on the ovarian carcinoma dataset

*K*	Dendrix	*Time*(*s*)	$r_{pe}(\%)$
2	**MYC CCNE1**	364.3	100.0
3	**MYC CCNE1** *NINJ2*	299.3	66.7
4	**MYC CCNE1** *ABCC10 NINJ2*	300.4	50.0
5	**MYC CCNE1 COL5A3** *ABCC10 NINJ2*	268.5	60.0
6	**MYC CCNE1 COL5A3** *ABCC10 NINJ2 MYH4*	282.8	50.0
7	**MYC CCNE1 COL5A3** *ABCC10 NINJ2 KIAA1012 MYH4*	261.9	42.9
8	**MYC CCNE1 COL5A3** *ABCC10 NINJ2 KIAA1012 MYH4 PEG3*	269.4	37.5

*K*	GA	*Time*(*s*)	$r_{pe}(\%)$
2	**MYC CCNE1**	8.2	100.0
3	**MYC CCNE1** *NINJ2*	10.3	66.7
4	**MYC CCNE1** *MYH4 NINJ2*	12.2	50.0
5	**MYC CCNE1 COL5A3** *ABCC10 NINJ2*	15.5	60.0
6	**MYC CCNE1 COL5A3** *ABCC10 NINJ2 MYH4*	16.2	50.0
7	**MYC CCNE1 COL5A3** *ABCC10 NINJ2 PEG3 MYH4*	18.6	42.9
8	**MYC CCNE1 COL5A3** *ABCC10 NINJ2 PEG3 MYH4 KIAA1012*	21.3	37.5

*K*	iMCMC	*Time*(*s*)	$r_{pe}(\%)$
2	*KRAS PPP2R2A*	–	–
3	**MYC CCNE1** RAD52	–	–

*K*	PGA-MWS	*Time*(*s*)	$r_{pe}(\%)$
2	**MYC CCNE1**	45.0	100.0
3	**MYC CCNE1** * NINJ2*	137.0	66.7
4	**MYC CCNE1** * MACF1 NINJ2*	235.0	50.0
5	**MYC CCNE1** *MACF1 NINJ2 BRD4*	722.0	40.0
6	**MYC CCNE1** *MACF1 NINJ2 BRD4 RYR2*	813.0	33.3
7	**MYC CCNE1** *MACF1 NINJ2 BRD4 KIF26B ZDHHC11*	1145.0	28.6
8	**MYC CCNE1** *MACF1 NINJ2 BRD4 KIF26B ZDHHC11 USH2A*	741.0	25.0

*K*	CGA-MWS	*Time*(*s*)	$r_{pe}(\%)$
2	**MYC CCNE1**	4.5	100.0
3	**MYC CCNE1** *NINJ2*	5.2	66.7
4	**MYC CCNE1** *MACF1 NINJ2*	5.5	50.0
5	**MYC CCNE1** *MACF1 NINJ2 NF1*	7.0	40.0
6	**MYC CCNE1** *MACF1 NINJ2 BRD4 LRP2*	8.2	33.3
7	**MYC CCNE1** *MACF1 NINJ2 BRD4 ZDHHC11 LRP2*	9.5	28.6
8	**MYC CCNE1** *MACF1 NINJ2 BRD4 USH2A KIF26B TBP*	10.3	25.0

*K*	CPGA	*Time*(*s*)	$r_{pe}(\%)$
2	**MYC CCNE1**	257.9	100.0
3	**MYC CCNE1 KRAS**	416.4	100.0
4	**MYC CCNE1 KRAS** *TERT*	626.8	75.0
5	**MYC CCNE1 KRAS STK11** *NF1*	1206.4	80.0
6	**MYC CCNE1 KRAS STK11 PIK3CA** *NF1*	756.6	83.3
7	**MYC CCNE1 KRAS STK11 PIK3CA PTEN** *NF1*	1167.8	85.7
8	**MYC CCNE1 KRAS STK11 PIK3CA PTEN** *CDH1 TERT*	1208.4	75.0

*K*	CPGA-SMCMN	*Time*(*s*)	$r_{pe}(\%)$
2	**MYC KRAS**	529.8	100.0
3	**MYC CCNE1 KRAS**	762.8	100.0
4	**MYC CCNE1 KRAS PIK3CA**	1383.6	100.0
5	**MYC CCNE1 KRAS PIK3CA PTEN**	1645.0	100.0
6	**MYC CCNE1 KRAS PIK3CA PTEN STK11**	867.4	100.0
7	**MYC CCNE1 KRAS PIK3CA PTEN STK11** *CTNNB1*	2544.8	85.7
8	**MYC CCNE1 KRAS PIK3CA PTEN STK11** * CTNNB1 TERT*	1429.7	75.0

Bold indicate that the genes are enriched in the same biological signaling pathway

When *K* is greater than 3, methods CPGA and CPGA-SMCMN can produce superior gene sets to the other methods in terms of pathway enrichment. In particular, when *K* = 3–6, all of the genes obtained by method CPGA-SMCMN are engaging in the *PI3K-Akt* signaling pathway. When *K* = 7 and 8, although *CTNNB1* and *TERT* are not involved in the *PI3K-Akt* signaling pathway together with other genes, they are critical OVCA related genes. *CTNNB1* mutations in the ovary are characteristic features of ovarian carcinomas [31]. The methylation of *TERT* is one of the important characteristics of ovarian carcinomas [32]. In addition, genes (*KRAS, PIK3CA, PTEN, STK11*) are also engaging in the *mTOR* signaling pathway (Fig. 6). It is acknowledged that the alterations in genes associated with the *PI3K/AKT/mTOR* pathway are commonly found in ovarian cancer [33].

**Fig. 7:**
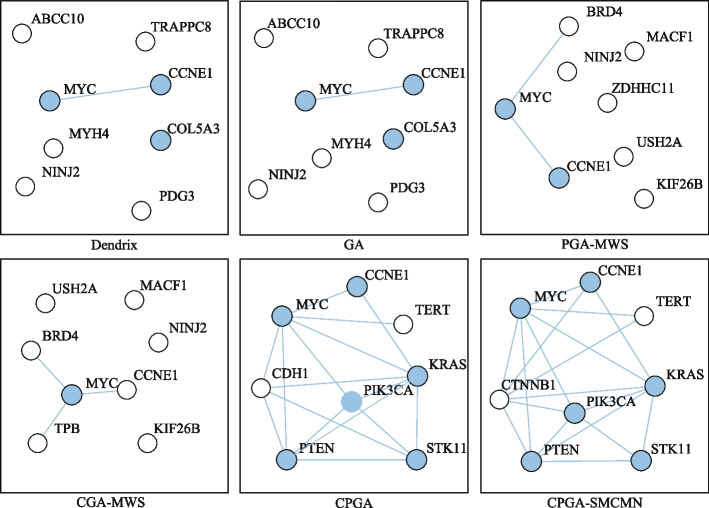
Connectivity of genes in the PPI network (OVCA dataset, *K* = 8)

**Fig. 8:**
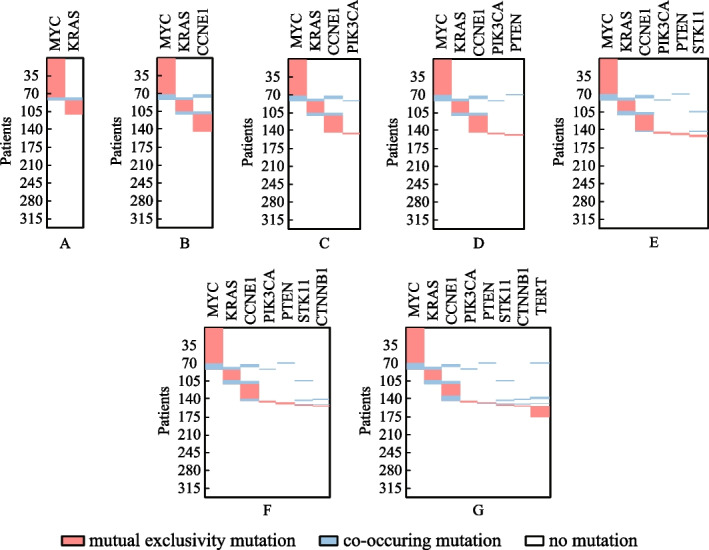
The coverage and mutual exclusivity of the gene sets detected by the CPGA-SMCMN method (OVCA dataset)

From Table 6, it can be observed that the running time increases comparative to that spent in the GBM database, for the OVCA dataset has much more samples and genes than the GBM dataset. Except for the Dendrix method, the efficiency of other methods are affected by the size of identified gene set *K*. In Fig. 7, the connectivity of genes detected by different methods in the PPI network is displayed, where *K* = 8. The gene sets found by methods CPGA and CPGA-SMCMN still exhibit better connectivity than those acquired by the other methods. Since they all have *p-values* less than 0.005, they are statistically significant. Figure 8 illustrates the coverage and mutual exclusivity of the detected gene sets, where *K* ranges from 2 to 8. At least one-third patients are covered by each gene set. Gene *MYC* mutates in more than a quarter of patients. It has been demonstrated that ovarian cancer cells highly rely on *MYC* for maintaining their oncogenic growth, and *MYC* is a therapeutic target for ovarian cancer [34]. In addition, some genes with low mutation frequency are also contained in the detected gene sets. For example, gene *PIK3CA* mutates in 5 patients, gene *PTEN* mutates in 6 samples, and gene *STK11* mutates in 8 samples.

#### Thyroid carcinoma

**Fig. 9:**
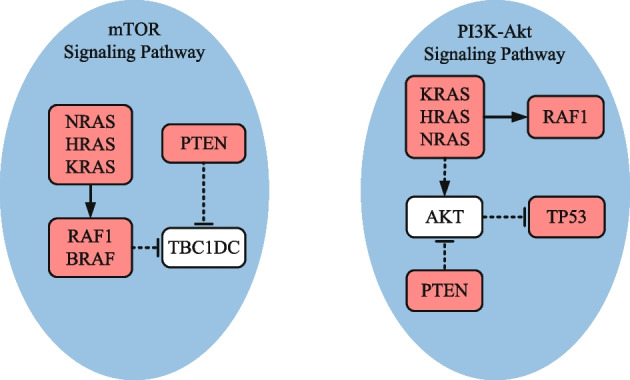
Biological pathways enriched with the genes detected by method CPGA-SMCMN (THCA dataset)

In Table 7, the identified gene sets and execution time are compared based on the THCA dataset, where *K* = 2–8. Since Zhang et al. [12] and Zheng et al. [13] do not provide the results of methods iMCMC and MOGA on this dataset, they were not compared. It can be clearly observed that the results acquired by methods CPGA and CPGA-SMCMN are close in the number of enriched genes, and manifest much superior enrichment performance to those detected with other methods. As displayed in Fig. 9, the genes recognized by the CPGA-SMCMN method involve in two crucial signaling pathways, i.e., the *mTOR* signaling pathway and the *PI3K-Akt* one. The overactivation of the *PI3K/Akt/mTOR* pathway plays a significant role in the pathogenesis of medullary thyroid cancer [35]. Though three genes, i.e., *RET*, *CDKN2A*, and *JAK2*, are not enriched in a cancer related pathway with other detected genes, they are believed to have a close relationship with thyroid carcinoma. *RET* alterations have been identified in diverse thyroid cancer subtypes, and its fusions have been demonstrated to be a common oncogenic driver event of papillary thyroid carcinoma [36]. The increased expression of *CDKN2A* gene product is associated with thyroid cancer progression [37]. It has been reported recently that gene *JAK2* may be a latent target of oridonin in the treatment of thyroid cancer [38]. The running time exhibited in Table 7 demonstrates that all of the methods can solve the problem in feasible time.

**Table 7:** Comparisons of experimental results on the thyroid carcinoma dataset

*K*	Dendrix	*Time*(*s*)	$r_{pe}(\%)$
2	**BRAF NRAS**	358.9	100.0
3	**BRAF NRAS HRAS**	364.6	100.0
4	**BRAF NRAS HRAS PTEN**	380.0	100.0
5	**BRAF NRAS HRAS** * LIPJ CNTLN*	360.3	60.0
6	**BRAF NRAS HRAS** *DOK6 CNTLN GLUD1*	380.3	50.0
7	**BRAF NRAS HRAS** *LIPJ CNTLN MYO1C SLC25A45*	359.8	42.6
8	**BRAF NRAS HRAS** *ZCCHC2 CNTLN CFAP70 SUV39H2 SLC25A45*	375.6	37.5

*K*	GA	*Time*(*s*)	$r_{pe}(\%)$
2	**BRAF NRAS**	4.3	100.0
3	**BRAF NRAS HRAS**	5.6	100.0
4	**BRAF NRAS HRAS** *CCSER2*	6.1	75.0
5	**BRAF NRAS HRAS PTEN** *CNTLN*	8.0	80.0
6	**BRAF NRAS HRAS PTEN** *ZCCHC2 CNTLN*	8.9	66.7
7	**BRAF NRAS HRAS PTEN** * ZCCHC2 CNTLN DOCK6*	9.7	57.1
8	**BRAF NRAS HRAS PTEN KRAS** * ZCCHC2 CNTLN DOCK6*	10.2	62.5

*K*	PGA-MWS	*Time*(*s*)	$r_{pe}(\%)$
2	**BRAF NRAS**	45.0	100.0
3	**BRAF NRAS HRAS**	89.0	100.0
4	**BRAF NRAS PTEN** * GTPBP4*	169.0	75.0
5	**BRAF NRAS HRAS PTEN** *GTPBP4*	435.0	80.0
6	**BRAF NRAS HRAS PTEN** *GTPBP4 TAF18*	648.0	66.7
7	**BRAF NRAS HRAS PTEN** *GTPBP4 VAPA CEP120*	828.0	57.1
8	**BRAF NRAS HRAS PTEN** * GTPBP4 CNTLN DOCK6 SLC25A45*	1026.0	50.0

*K*	CGA-MWS	*Time*(*s*)	$r_{pe}(\%)$
2	**BRAF NRAS**	1.9	100.0
3	**BRAF NRAS HRAS**	2.4	100.0
4	**BRAF NRAS HRAS PTEN**	2.6	100.0
5	**BRAF NRAS HRAS PTEN** * CNTLN*	3.5	80.0
6	**BRAF NRAS HRAS** * CNTLN TG PRKG1*	4.0	50.0
7	**BRAF NRAS HRAS** * CNTLN TG SYCE1 PRKG1*	4.4	42.6
8	**BRAF NRAS HRAS** * CNTLN TG SYCE1 DOCK6 PRKG1*	5.4	37.5

*K*	CPGA	*Time*(*s*)	$r_{pe}(\%)$
2	**BRAF NRAS**	68.1	100.0
3	**BRAF NRAS HRAS**	66.6	100.0
4	**BRAF NRAS HRAS PTEN**	60.1	100.0
5	**BRAF NRAS HRAS PTEN** * RET*	116.2	80.0
6	**BRAF NRAS HRAS PTEN KRAS** * RET*	128.2	83.3
7	**BRAF NRAS HRAS PTEN KRAS RAF1** * RET*	109.2	85.7
8	**BRAF NRAS HRAS PTEN KRAS RAF1** * RET JAK2*	103.8	75.0

*K*	CPGA-SMCMN	*Time*(*s*)	$r_{pe}(\%)$
2	**BRAF NRAS**	255.8	100.0
3	**BRAF NRAS HRAS**	199.5	100.0
4	**BRAF HRAS NRAS PTEN**	186.8	100.0
5	**BRAF HRAS NRAS PTEN KRAS**	165.4	100.0
6	**BRAF HRAS NRAS PTEN KRAS** * RET*	165.0	83.3
7	**BRAF HRAS NRAS PTEN KRAS RAF1** * CDKN2A*	205.8	85.7
8	**BRAF HRAS NRAS PTEN KRAS RAF1** * RET JAK2*	136.7	75.0

Bold indicate that the genes are enriched in the same biological signaling pathway

**Fig. 10:**
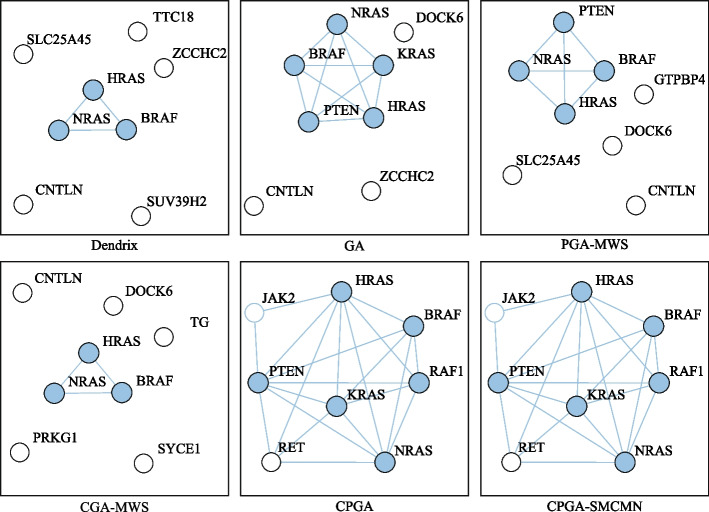
Connectivity of genes in the PPI network (THCA dataset, *K* = 8)

**Fig. 11:**
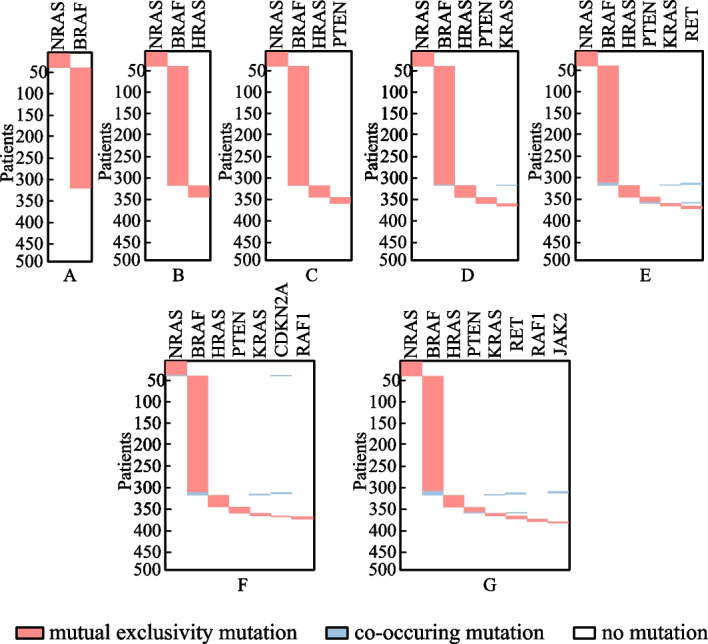
The coverage and mutual exclusivity of the gene sets detected by the CPGA-SMCMN method (THCA dataset)

Figure 10 shows the connectivity of genes identified by different methods in the PPI network with *K* = 8. The genes recognized by methods CPGA-SMCMN and CPGA are absolutely the same, and present stronger connectivity than the genes acquired with other methods. Each of the eight gene sets detected by method CPGA-SMCMN has a *p-value* of less than 0.005, hence they are statistically significant. The coverage and mutual exclusivity of them are illustrated in Fig. 11. It can be discovered that at least two-thirds of patients are covered by each gene set, and gene *BRAF* does a great contribution to the coverage. There are about 45% of sporadic papillary thyroid cancers have genetic variation in this gene [39]. Furthermore, a low-frequency gene *RAF1*, mutating in 3 patients, was recognized by method CPGA-SMCMN and was enriched in the *mTOR* and the *PI3K-Akt* signaling pathways with other identified genes.

## Discussion

The problem of identifying cancer driver pathways has drawn great attention in the area of studying cancers. In this article, the relative hamming distance RHD is devised for calculating the distance between a gene and a gene set, and a new measurement of mutual exclusivity is put forward based on RHD to exclude the gene sets having an “inclusion” relationship. A parameter-free identification model SMCMN is proposed by ascertaining a submatrix having maximum coverage, mutual exclusivity and network connectivity. Furthermore, a partheno-genetic algorithm is presented by introducing gene clustering based operators for initializing and recombining individuals.

The performance of algorithm CPGA is closely related with a pair of artificial parameters, i.e., $\mu$ and $\nu$, whose values were determined with abundant pre-experiments. How to eliminate them by combining different omics data will be studied in the future. In addition, during the process of experiments, it is confirmed that the execution efficiency of method CPGA-SMCMN decreases obviously with the increase of gene number. The improvement of execution efficiency will also become a focus of future studies.

## Figures and Tables

**Fig. 12:**
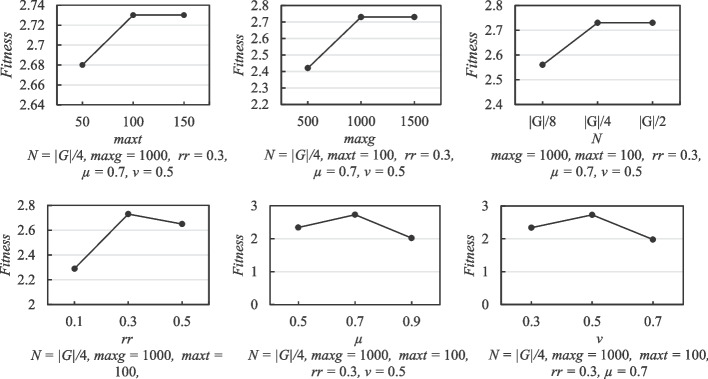
The comparing experimental results with different *maxg*, *maxt*, *N*, *rr*, $\mu$ and $\nu$(OVCA dataset, *K* = 8)

## Data Availability

The source code and datasets generated or analysed during the current study are available in https://github.com/gxnubioinfo/CPGA-SMCMN.git.

## Appendix A: Experimental results under different *maxg*, *maxt*, *N*, *rr*, $\mu$ and $\nu$

Figure 12 demonstrates the average *Fitness* obtained for solving the most complex OVCA dataset under different combinations of parameters *maxg*, *maxt*, *N*, *rr*, $\mu$, and $\nu$. Ten runs were performed for each group of parameters, and the average over ten runs was calculated and displayed. Since *Fitness* varies between a narrow range or remains unchanged under different parameter settings, the middle values of them are chosen for the trade-off between *Fitness* and execution efficiency, i.e., *maxg* = 1000, *maxt* = 100, *N* = $\frac{|G|}{4}$, *rr* = 0.3, $\mu$ = 0.7, $\nu$ = 0.5.
